# Mechanisms of Aβ Clearance and Degradation by Glial Cells

**DOI:** 10.3389/fnagi.2016.00160

**Published:** 2016-07-05

**Authors:** Miriam Ries, Magdalena Sastre

**Affiliations:** Division of Brain Sciences, Imperial College London, Hammersmith HospitalLondon, UK

**Keywords:** astrocytes, microglia, amyloid-β, Alzheimer’s disease, proteases, phagocytosis

## Abstract

Glial cells have a variety of functions in the brain, ranging from immune defense against external and endogenous hazardous stimuli, regulation of synaptic formation, calcium homeostasis, and metabolic support for neurons. Their dysregulation can contribute to the development of neurodegenerative disorders, including Alzheimer’s disease (AD). One of the most important functions of glial cells in AD is the regulation of Amyloid-β (Aβ) levels in the brain. Microglia and astrocytes have been reported to play a central role as moderators of Aβ clearance and degradation. The mechanisms of Aβ degradation by glial cells include the production of proteases, including neprilysin, the insulin degrading enzyme, and the endothelin-converting enzymes, able to hydrolyse Aβ at different cleavage sites. Besides these enzymes, other proteases have been described to have some role in Aβ elimination, such as plasminogen activators, angiotensin-converting enzyme, and matrix metalloproteinases. Other relevant mediators that are released by glial cells are extracellular chaperones, involved in the clearance of Aβ alone or in association with receptors/transporters that facilitate their exit to the blood circulation. These include apolipoproteins, α2macroglobulin, and α1-antichymotrypsin. Finally, astrocytes and microglia have an essential role in phagocytosing Aβ, in many cases via a number of receptors that are expressed on their surface. In this review, we examine all of these mechanisms, providing an update on the latest research in this field.

## Introduction

The pathogenesis of Alzheimer’s disease (AD) has been associated with the presence of extracellular amyloid-β peptide (Aβ) aggregates, forming neuritic plaques, as well as intra-neuronal Aβ (Gouras et al., 2005; Blair et al., 2014), probably due to alterations in the mechanisms of generation and/or clearance of amyloid in the brain during aging. There is evidence of an increase in the expression of β-APP cleaving enzyme-1 (BACE1), the enzyme responsible for the cleavage of the amyloid precursor protein (APP) in the amyloidogenic pathway, in sporadic AD cases (Holsinger et al., 2002; Yang et al., 2003). In addition, the dysregulation of the systems involved in the clearance and degradation of Aβ has generated a lot of interest in the last decade, particularly their effect on the accumulation of Aβ in the blood vessel walls, leading to cerebral amyloid angiopathy (Love, 2004).

A great number of reviews have examined the main mechanisms of Aβ clearance and degradation (Bates et al., 2009; Saido and Leissring, 2012; Yoon and Jo, 2012; Tarasoff-Conway et al., 2015). These include Aβ proteases, which are enzymes that degrade or cleave Aβ into smaller fragments. Other proteins with a crucial role in Aβ clearance are apolipoprotein E (ApoE) and α2-macroglobulin (α2-M); they interact with transporters including low-density lipoprotein receptor-related protein 1 (LRP1) receptors, very low-density lipoprotein receptor (VLDLR), and P-glycoprotein, localized in astrocytes and on the abluminal side of the cerebral endothelium, where they facilitate the transport of Aβ across the blood brain barrier (BBB) into the blood circulation (Deane et al., 2009). Genetic mutations resulting in loss of function of those proteins have demonstrated their importance in disease progression, particularly in late onset AD, although there are indications of alterations of some of the Aβ proteases by environmental factors (**Table 1**). Experimental data have suggested that Aβ and solutes can normally also be cleared along the lymphatic drainage pathways within the basement membranes of capillaries and arteries (Carare et al., 2008; Tarasoff-Conway et al., 2015). Lastly, another way to eliminate Aβ from the brain is by the uptake and phagocytosis of Aβ by cells such as microglia, astrocytes, and macrophages.

**Table 1:** Genetic vs environmental factors affecting Aβ clearance.

Genetic factors	Environmental factors
ApoE mutations (Verghese et al., 2013)	Metal ions affect expression of IDE, NEP and metalloproteases (Sastre et al., 2015)
ApoJ mutations (Harold et al., 2009)	Insulin/diabetes affects IDE levels (Steneberg et al., 2013)
Presenilin mutations affect microglia function (Farfara et al., 2011)	Oxidative stress regulates IDE (Stocker and Keaney, 2004)
TREM-2 mutations (Kleinberger et al., 2014)	

**FIGURE 1:**
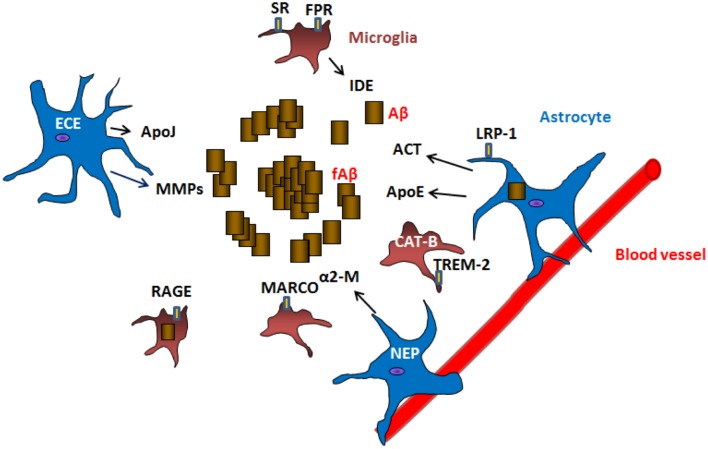
**Mechanisms of Aβ clearance by glial cells**. Astrocytes and microglia can produce Aβ degrading proteases neprilysin (NEP), endothelin-converting enzyme (ECE), insulin degrading enzyme (IDE), matrix metalloproteases (MMPs), cathepsin B (CAT-B) as well as chaperones apolipoprotein E (ApoE), apolipoprotein J (ApoJ), α-2 macroglobulin (α2-M), α1-antichymotrypsin (ACT) involved in the clearance of Aβ. Receptors located in the surface of glial cells such as lipoprotein receptor-related protein 1 (LRP-1), scavenger receptors (SR), formyl peptide receptors (FPR), macrophage receptor with collagenous structure (MARCO), receptor for advanced glycation end products (RAGE) and triggering receptor expressed on myeloid cells 2 (TREM-2) are involved in the uptake and clearance of Aβ (receptor mediated endocytosis). Astrocytes are also connected to blood vessels, where they are implicated in the draining of Aβ and other products out of the brain.

Many of the mechanisms mentioned above are mediated by glial cells. Here, we review how glial cells are mediators of Aβ removal from the brain (schematic **Figure 1**).

## Aβ Degrading Proteases

Many of the proteins involved in the enzymatic degradation of Aβ are produced by glial cells. These proteases cleave at different sites within the Aβ sequence, resulting in different enzymatic products (Nalivaeva et al., 2012). These include:

### Metalloendopeptidases

Neprilysin (NEP), the insulin degrading enzyme (IDE) and endothelin-converting enzymes-1 and -2 (ECE1 and ECE2) (Eckman et al., 2001, 2003) are metalloendopeptidases particularly involved in the degradation of monomeric Aβ species (although neprilysin can also hydrolyse oligomeric forms). ECEs are expressed in neurons, endothelial cells (Naidoo et al., 2004), and astrocytes (Nakagomi et al., 2000) and primarily degrade Aβ intracellularly (Eckman et al., 2003). NEP is mainly expressed in pre-synaptic terminals of neurons (Fukami et al., 2002) but can also be found in activated astrocytes (Fisk et al., 2007; Yamamoto et al., 2014) and microglia (Hickman et al., 2008). In the brain, IDE is synthesized and secreted by neurons, oligodendrocytes, and microglia (Bernstein et al., 2008), where it is released via exosomes (Tamboli et al., 2010) especially acting on extracellular Aβ deposits. IDE is unequally expressed in human brain, majorly found in hypothalamic neurons and in hippocampus, cerebellum, and brain stem (Bernstein et al., 2008), coinciding with the location of insulin receptors in the brain.

The relevance of NEP and IDE has been proven in knockout models, whereby mice lacking those enzymes crossed with APP transgenic models show increased Aβ deposition (Iwata et al., 2001; Farris et al., 2003) in the brain. Conversely, overexpression of these proteases has been shown to reduce Aβ load (Leissring et al., 2003).

The expression of NEP and IDE in glial cells has been found to change depending on the stage of the disease. *In vitro* and *in vivo* studies have shown that IDE and ECE are up-regulated in response to increasing levels of brain Aβ (Zhao et al., 2007), while NEP has been found reduced in AD brains (Wang et al., 2010).

### Plasminogen Activators and ACE

Other enzymes also involved in Aβ degradation, but whose relevance depends on the degree of Aβ pathology, are plasminogen activators and angiotensin-converting enzyme (ACE) (Hu et al., 2001), which are more effective for aggregated Aβ rather than regulating steady-state Aβ levels (Saido and Leissring, 2012). While ACE is mainly neuronal, tissue plasminogen activator (tPA) is synthesized by neurons and microglial cells (Melchor and Strickland, 2005).

### Matrix Metalloproteinases (MMPs)

Matrix metalloproteinases, known to be expressed and secreted by astrocytes, play a role in the extracellular degradation of both monomeric and fibrillar forms of Aβ (Yan et al., 2006). Astrocytes surrounding amyloid plaques show enhanced expression of MMP-2 and MMP-9 in aged APP/presenilin 1 mice. Moreover, astrocyte-conditioned medium (ACM) degraded Aβ, lowering its levels and producing several fragments after incubation with synthetic human Aβ(1–40) and Aβ(1–42). This activity was attenuated with specific inhibitors of MMP-2 and -9, as well as in ACM derived from mmp-2 or -9 knockout (KO) mice (Yin et al., 2006).

### Lysosomal Peptidases

It has been described that after being phagocytosed by microglia, Aβ can be degraded by cathepsin B (CAT-B) (Nakanishi, 2003; Halle et al., 2008). This enzyme is able to reduce longer forms of Aβ into shorter less toxic species, such as Aβ38. In addition, ECE-2 can be found expressed in endosomes/lysosomes where it can mediate the degradation of Aβ (Pacheco-Quinto and Eckman, 2013).

## Proteasomal Degradation

The ubiquitin proteasome system (UPS) is a mechanism of protein degradation which selectively targets individual proteins, including short-lived, damaged, or defectively folded proteins (Rock et al., 1994; Lilienbaum, 2013). Before a protein is degraded by the proteasome, the ubiquitin (Ub) system selects the protein target by the conjugation of Ub carried out by the serial activity of several enzymes (E1–E3) along the pathway. It has been shown that the proteasomal degradation machinery is capable of cleaving Aβ(1–42) peptides in a dose-dependent manner, without significantly affecting the overall catalytic function of the proteasome (Zhao and Yang, 2010). In addition, it was shown that E2 conjugating enzymes, E3 ligases, and de-ubiquitinating enzymes play a pivotal role in the proteasomal degradation of Aβ (Hong et al., 2014). Interestingly, it seems that the UPS system in glial cells is more efficient at degrading aggregated proteins compared with neurons (Jansen et al., 2014), and this could explain why they do not contain protein aggregates. However, in neurodegenerative diseases the UPS system in glia could become dysfunctional and contribute to the progression of the disease.

## Autophagic Degradation

The autophagy pathway is critical for the turnover of cell organelles and degradation of aggregated proteins in cells under stress. It was hypothesized that a defective clearance of Aβ-generating autophagic vacuoles creates conditions favorable for Aβ accumulation in AD and this is supported by data indicating that increasing autophagy by rapamycin reduces amyloid burden *in vivo* (Nixon, 2007). The role of autophagy in the degradation of Aβ has not been investigated in glial cells until recently. Cho and colleagues reported the importance of autophagy in the clearance of extracellular Aβ fibrils by microglia and in the regulation of the Aβ-induced NLRP3 (NLR Family, Pyrin Domain Containing 3) inflammasome using microglia from atg7 knockout mice and *in vitro* cultures (Cho et al., 2014). Interestingly, microglia isolated from human AD brains show significantly reduced beclin 1 and retromer protein levels (Lucin et al., 2013). In addition, astrocytes from transgenic AD models showed strong expression of microtubule-associated protein light chain 3 (LC3), and autophagy seems to be involved in Aβ internalization by those cells (Pomilio et al., 2016), providing a link between autophagy and phagocytosis.

## Aβ Clearance by Extracellular Chaperones

These include proteins that bind Aβ in plasma and cerebrospinal fluid (CSF), such as albumin, α2M, α1-antichymotrypsin (ACT), serum amyloid P component (SAP), complement proteins, transthyretin, apoferritin, apolipoproteins, and lipoproteins (Bates et al., 2009), which are essential because they modulate the formation of Aβ fibrils and mediate the interaction of Aβ with LRP-1 receptors in astrocytes.

### Apolipoprotein E (ApoE)

It is the major risk factor for late onset AD. It is produced primarily by astrocytes in the brain and has been implicated in the degradation of Aβ in these cells (Koistinaho et al., 2004), although it can be produced by microglia and neurons in response to stimuli from glial cells (Saura et al., 2003; Harris et al., 2004). It has been shown that ApoE binds Aβ, and this association is more efficient with the ApoE2 and E3 isoforms than with ApoE4; these complexes are thought to influence both seeding of fibrillar Aβ and transport of soluble Aβ (Wildsmith et al., 2013). However, some recent controversial reports indicate that ApoE does not bind Aβ but competes with Aβ for binding to LRP-1 in astrocytes, and this could impact Aβ clearance by glia and across the blood–brain barrier (BBB) (Verghese et al., 2013). However, it is clear that ApoE has a role on Aβ clearance because bexarotene, which enhances ApoE expression, clears Aβ and improves cognition in mice (Cramer et al., 2012).

### Apolipoprotein J (ApoJ or Clusterin)

Apolipoprotein J has been shown to interact with Aβ and alters its ability to form fibrils as well as modifying Aβ-mediated neurotoxicity. Similarly to ApoE, ApoJ is also produced in astrocytes. ApoJ is known to facilitate the transport of Aβ and hence the clearance across the BBB through the megalin/LRP-2 receptor. Upon exposure to Aβ combined with ApoE, ApoJ, ACT and a combination of SAP and complement C1q, a clear reduction in astrocytic but not microglial oligomeric Aβ uptake was observed (Mulder et al., 2014).

### α1-Antichymotrypsin (ACT)

α1-Antichymotrypsin is a serine protease inhibitor that has been reported to bind Aβ and is overexpressed in the brain of AD patients (Abraham, 2001). ACT has been shown to be produced in astrocytes (Abraham et al., 1989) and its expression is regulated by proinflammatory cytokines including interleukin (IL)-1, oncostatin M (OSM), and complexes of IL-6, soluble IL-6 receptors and transcriptional regulators such as nuclear factor 1-X and activator protein 1 (AP-1; Gopalan et al., 2006a,b).

### α2-Macroglobulin (α2-M)

α2-Macroglobulin is a matrix metalloproteinase inhibitor that is also released by glial cells, in particular perivascular astrocytes (Cucullo et al., 2003). Microinjection of clusterin and α2-M into the hippocampus of rat brains were found to prevent Aβ42-induced learning and memory impairments and reduce Aβ42-induced glial inflammation and neuronal degeneration (Cascella et al., 2013), suppressing oligomer cytotoxicity. α2-M can act as a ligand for LRP-1 (Kanekiyo et al., 2014), promoting increased neurite outgrowth in primary sensory neurons (Yamauchi et al., 2013).

## Aβ Internalisation

An important mechanism of Aβ clearance from the brain is its uptake by glial cells. Aβ can be internalized by microglia, astrocytes, and other immune cells such as macrophages.

### Pinocytosis

Soluble Aβ can be cleared by microglia through fluid phase pinocytosis (Mandrekar et al., 2009), with spontaneous formation and internalization of pinosomes from membrane ruﬄes. Furthermore, soluble Aβ(1–42) is able to induce its pinocytic self-uptake by stimulating the P2Y4 receptor and the PI3kinase/Akt cascade through autocrine ATP signaling on microglia (Li et al., 2013).

### Phagocytosis

It is widely accepted that microglia phagocytose fibrillar Aβ, particularly more vigorously when bound by the C3b complement system (Lee and Landreth, 2010). Interestingly, the induction of microglial phagocytosis by fibrillar Aβ is attenuated by oligomeric Aβ (Pan et al., 2011). Astrocytes can also endocytose monomeric and oligomeric Aβ through actin regulation (Lee et al., 2015). In addition, there is evidence that astrocytes are able to phagocytose neurons containing Aβ (Nagele et al., 2003).

### Receptor-Mediated Endocytosis

Oligomeric and fibrillar Aβ are primarily internalized though receptor-mediated endocytosis, via a number of receptors that are expressed on the surface of microglia and astrocytes:

#### Scavenger Receptors (SR)

Scavenger receptor type-A (SR-A), type B1 (SR-B1), CD36 and CD40 are able to bind and mediate the endocytosis of oligomeric and fibrillar Aβ (Paresce et al., 1996; Coraci et al., 2002; Husemann et al., 2002; El Khoury et al., 2003; Yang et al., 2011). SR-As can act in conjunction with other receptors, such as complement receptor 3 (also known as Mac-1/CD11b) to promote the uptake of fibrillar Aβ in microglia (Fu et al., 2012). On the other hand, the class B scavenger receptors CD36/SR-BII are not involved in oligomeric Aβ clearance, but can affect the recruitment and activation of microglia in response to fibrillar Aβ (Coraci et al., 2002; El Khoury et al., 2003). Fibrillar Aβ also acts as a scaffold for the assembly of a receptor complex consisting of CD36, alpha6beta1-integrin, and CD47 (Bamberger et al., 2003). Besides microglia, some types of SRs are expressed by astrocytes, including SR-BI and SR-MARCO (macrophage receptor with collagenous structure; Alarcon et al., 2005).

#### Toll-Like Receptors (TLR)

Toll-like receptors are involved in the microglial clearance of monomeric, oligomeric, and fibrillar Aβ (Tahara et al., 2006; Reed-Geaghan et al., 2009). TLR2 and TLR4 are directly involved in the microglial phagocytic response to Aβ, or indirectly with other receptors, as in the case of TLR9 activation by Aβ through the upregulation of formyl peptide receptor-2 (FPR2; Iribarren et al., 2005). The phagocytosis of fibrillar Aβ(1–42) by microglia can also be mediated through the LPS receptor CD14, a co-receptor of the functional TLR complex (Liu et al., 2005). Studies performed in TLR2 and TLR4 knockout mice have confirmed the importance of these receptors in Aβ clearance, showing increased Aβ deposition (Tahara et al., 2006; Richard et al., 2008; Birch et al., 2014). Some TLRs are also expressed in astrocytes and respond to TLR activators by secreting pro-inflammatory molecules (Gorina et al., 2009; van Noort and Bsibsi, 2009; Trudler et al., 2010).

#### Receptor for Advanced Glycation End Products (RAGE)

Although RAGE receptors have been mainly involved in mediating the inflammatory cascade in activated microglia (Solito and Sastre, 2012), their role in Aβ phagocytosis in astrocytes has been recently demonstrated when blocking these receptors with specific antibodies (Jones et al., 2013).

#### Formyl Peptide Receptors (FPR)

Formyl peptide receptors are a group of seven-transmembrane G protein coupled receptors (Le et al., 2002) and are expressed in neurons, microglia and astrocytes (Panaro et al., 2007; Braun et al., 2011). There is evidence that the FPRL1/FPR2 subtype binds to Aβ(1–42) and activates microglial internalization of the Aβ/FPRL1 complex in a PLD dependent-manner in microglia (Le et al., 2001; Brandenburg et al., 2008) and astrocytes (Brandenburg et al., 2008). In addition, both RAGE and SR-MARCO are known to form complexes with FPRL1/FPR2 in the presence of Aβ, initiating microglial signaling cascades in response to Aβ (Brandenburg et al., 2010; Slowik et al., 2012).

#### Fc Receptors (FcRs)

Fc receptors are expressed in microglia, astrocytes, oligodendrocytes, and neurons (Okun et al., 2010). Peress and colleagues first reported FcγRI, FcγRII, and FcγRIII immunoreactivity in senile plaques and on ramified microglia throughout the cortex and white matter of healthy controls and AD patients (Peress et al., 1993). The FcRs have been shown to mediate Aβ clearance in the presence of anti-Aβ antibodies (Bard et al., 2000; Wilcock et al., 2003), as observed in Aβ immunization therapies (Bacskai et al., 2002). The degree of involvement of Fc receptors in the clearance of Aβ bound by endogenous antibodies such as IgGs is currently not well elucidated (Doens and Fernández, 2014).

#### Triggering Receptor Expressed on Myeloid Cells 2 (TREM2)

Genetic variants of TREM2 receptors were recently identified as causing increased susceptibility to late onset AD. TREM2 can activate phagocytosis in microglia and reduce TLR-mediated signaling in macrophages (Klesney-Tait et al., 2006). Missense mutations associated with FTD and FTD-like syndrome have been shown to reduce TREM2 maturation and impair the phagocytic activity of TREM2-expressing cells (Kleinberger et al., 2014). More recently, it has been demonstrated that TREM2 is able to specifically sense fibrillar Aβ, activating microglial clustering around plaques, thereby limiting Aβ diffusion and subsequent toxicity (Wang et al., 2015, 2016).

#### Lipoprotein Receptor-Related Proteins (LRPs)

Lipoprotein receptor-related protein 1 (LRP1) is a large endocytic receptor for more than 40 ligands, including ApoE, α2-M and Aβ, and it is expressed by neurons, vascular cells and glial cells in the brain. Astrocytes take up Aβ through LRP1 either directly or indirectly in the presence of amyloid-associated protein ApoE, with perivascular astrocytes in AD brains found to contain both Aβ and ApoE (Utter et al., 2008; Kanekiyo et al., 2014). ApoE deficient astrocytes do not respond as well as wild type astrocytes to amyloid deposits, suggesting that ApoE is needed for astrocyte clearance of Aβ (Koistinaho et al., 2004). However, as indicated above, a recent paper has suggested that in fact ApoE may compete for the binding of Aβ to LRP-1. LRP-1 can also mediate Aβ phagocytosis in microglia, confirmed *in vitro* using LRP1 deficient cells (N’Songo et al., 2013). Furthermore, Aβ can be taken up when bound to LRP2 together with ApoJ and the megalin receptor (Zlokovic et al., 1996).

#### LGI3

The transmembrane protein leucine-rich glioma inactivated protein 3 co-localizes with Aβ at the astrocytic cell membrane (Kimura et al., 2007), and its downregulation reduces Aβ internalization by astrocytes (Okabayashi and Kimura, 2008).

## Astrocytes and the “Glymphatic” System

It has been recently shown that astrocytes may contribute to the clearance of debris from the brain thanks to their projections around blood vessels, creating a sort of network that drains Aβ and other products out of the brain. In vessels, astrocyte end feet appear to connect to the smooth muscle layer (Morris et al., 2014). High expression of the channel aquaporin 4 (AQP-4) at the astrocyte end feet is thought to help solute clearance due to its role in water transport (Igarashi et al., 2014). In fact, AQP-4 knock-out mice show hindered solute clearance including that of Aβ (Iliff and Nedergaard, 2013).

## Conclusion

Glial cells represent around 50% of the cells in the human brain (Azevedo et al., 2009). It is well established that in AD there is an up-regulation in the number and/or activation of microglia and astrocytes, associated with the deposition of Aβ. Although many studies have supported the notion that the activation of these glial cells may have detrimental effects due to the release of pro-inflammatory mediators such as certain cytokines and reactive oxygen species, there is evidence that supports their “protective” role by promoting the removal of Aβ. This function seems to be associated with a special and particular phenotype in microglia (formally known as M2 or alternatively activated) in contrast with the pro-inflammatory M1 state (Tang and Le, 2016). Therefore, many of the proteins that have been described in this review may not be expressed throughout life by glial cells, but their presence may depend on the activation status of those cells. This may also change during aging, when there is a dysregulation of glial function and these systems may become defective, contributing to the accumulation of Aβ in the brain.

A number of the studies reported here have outlined the difficulties of dissecting out each of these specific mechanisms of Aβ clearance only by using animal models with a specific deletion for one of those proteins, because many mechanisms of clearance are interconnected. Besides, some of these proteins have additional roles in the brain that are not directly related to the clearance of Aβ and may interfere with the interpretation of the results.

The therapeutic approaches targeting these clearance mechanisms have provided promising results, including the design of vectors carrying genes for NEP, for instance, or the discovery of drugs that enhance the synthesis of ApoE, showing reductions in Aβ deposition and improving cognitive impairments. Therefore, research in this field holds great potential for the development of new treatments to cure/stop the progression of AD.

## Author Contributions

MR wrote the mechanisms of Aβ internalization and organized the reference list and MS wrote the rest of the manuscript.

## Conflict of Interest Statement

The authors declare that the research was conducted in the absence of any commercial or financial relationships that could be construed as a potential conflict of interest.
